# Neotype Designation and Redescription of *Toumeyella liriodendri* (Gmelin) (Hemiptera: Coccoidea: Coccidae)

**DOI:** 10.1673/031.008.5601

**Published:** 2008-10-07

**Authors:** Takumasa Kondo, Douglas J. Williams

**Affiliations:** ^1^ Department of Entomology, University of California, I Shields Avenue, Davis, California 95616, U.S.A; ^2^ Department of Entomology, The Natural History Museum, London SW7 5BD, U.K

**Keywords:** *Coccus liriodendri* Gmelin, *Liriodendron tulipifera*, morphology, scale insect, tuliptree scale

## Abstract

In order to clarify the taxonomic status and to preserve the stability of the species, a neotype is designated for the tuliptree scale: *Coccus liriodendri* Gmelin (now *Toumeyella liriodendri*). The adult female of this scale insect is redescribed and illustrated from newly collected specimens in its native range and on its type host, the tulip tree, *Liriodendron tulipifera* L. (Magnoliales: Magoliaceae).

## Resumen

Para clarificar el estatus taxonómico y para preservar la estabilidad de la especie, un neotipo se designa para la escama del tulipán: *Coccus liriodendri* Gmelin (ahora *Toumeyella liriodendri*). La hembra adulta de la escama del tulipán se redescribe e ilustra en base de especímenes recientemente colectados en su ámbito nativo y en su hospedero tipo, el árbol del tulipán, *Liriodendron tulipifera*.

## Introduction

The tuliptree scale insect *Toumeyella liriodendri* (Gmelin) (Hemiptera: Coccoidea: Coccidae) was first described and named by Gmelin (1790) as *Coccus liriodendri* Gmelin. This work was published in three volumes and the description of the scale insect appeared in Volume 1, Part 4. This part covers Insecta and was published in 1790 (Soulsby 1933), not 1789 as usually quoted in scale insect literature. Gmelin's short entry in Latin can be translated as “Coccus of Liriodendron tulipifera. *Hamburg. Magaz.* 12. p. 1–24. It lives on Liriodendron tulipifera.” The reference cited by Gmelin contains an article by John Hill (Hill 1753) and is a German translation of an English article (Hill 1752). In the English article, Hill described a soft scale insect on a tuliptree grown at Goodwood, England, that had been imported from America some years previously. Although Hill did not name the insect, the description cited by Gmelin validated the name *Coccus liriodendri*. Hill's description of the insect was sufficient for Cockerell (1899) to place it as a species of *Lecanium* under the name *L. liriodendri* (Gmelin). There have been no reports of this species in England since Hill described it in 1753 and none of Gmelin's original specimens exist. Moreover, it is not listed as a British species by Boratynski and Williams (1964). The insect colony apparently died out long ago and no living specimens have been collected in Britain since.

The tuliptree, *Liriodendron tulipifera* L. (Magnoliales: Magoliaceae) (also known as yellow poplar), is one of two species of *Liriodendron* (Mabberley 1997) and is native to the Eastern U.S.A. The known range of the tuliptree encompasses most of Eastern North America, from Southern Canada to the middle of the Florida Peninsula and from the Atlantic coast to the Mississippi Valley, U.S.A. (Parks et al. 1994). The tree has a wide distribution in the U.S.A. because of its popularity as an ornamental tree.

*T. liriodendri* is an important pest of *L. tulipifera* and deciduous magnolias (Gill 1988; Hamon and Williams 1984; Kosztarab 1996). It produces large amounts of honeydew that induce sooty mould, and large populations will kill the host, particularly the tuliptree (Gill 1988). Seedlings of infested hosts are frequently killed by it (Kosztarab 1996). Since *T. liriodendri* was described, it has been recorded only from the U.S.A. where it follows a similar distribution pattern as its host. *T. liriodendri* is found in the Midwest and most states east of the Mississippi River (Gill 1988; Ben-Dov et al. 2005; Hamon & Williams 1984), and it also occurs in California (Armitage 1947; Gill 1988) and in Texas (Ben-Dov et al. 2005). It is common in the Northeastern U.S.A. where it occurs in every state south of New York and probably in Southeastern Canada (Kosztarab 1996). A full list of the twenty states where *T. liriodendri* occurs in the U.S.A. can be found in the scale insect database ScaleNet (Ben-Dov et al. 2005).

It was long suspected that the species described by Cook (1878) as *Lecanium tulipiferae* Cook was the same as the species described by Gmelin (Cockerell 1899). In fact, King (1902) stated that they were probably identical. Fernald (1903) synonymised the name *L. tulipiferae* with *C. liriodendri* and Sanders (1909) transferred the species to *Toumeyella* Cockerell.

As no original material of this species exists we here designate a neotype from specimens collected in Auburn, Alabama, U.S.A., that is within the area of its natural distribution, to clarify the taxonomic status of the species and for nomenclatural stability. The adult female was redescribed and illustrated previously (Williams and Kosztarab 1972; Gill 1988), but, because this species often varies in color and shape, we redescribe it and illustrate the adult female based on the neotype and specimens from the same population.

## Materials and Methods

Live specimens of *T. liriodendri* were collected from *L. tulipifera* in Auburn, Alabama. Specimens were slide-mounted using the method described by Williams and Granara de Willink (1992), except that xylene was used instead of clove oil. Morphological terminology follows mostly that of Hodgson (1994). Photographs of the population of the neotype were taken using a digital, Nikon COOLPIX 3100 camera (www.nikon.com), and were processed using the computer program Adobe Photoshop 5.0 (www.adobe.com).

## Specimen depositories

The material studied is deposited in the institutions listed below.

BME: Bohart Museum of Entomology, University of California, Davis, California, U.S.A.
BMNH: The Natural History Museum, London, England.
USNM: National Museum of Natural History Entomological Collection, Washington, D.C., U.S.A. (Coccoidea collection held at USDA, Beltsville, Maryland)
MNHN: Museum National d'Histoire Naturelle, Paris, France.

## Results and Discussion

**Figure 1.**
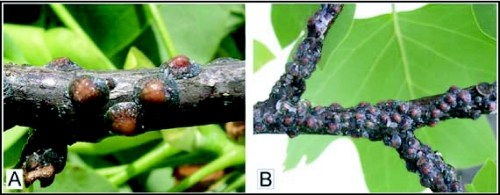
*Toumeyella liriodendri* on tuliptree. A, close-up of adult females; B, twig of the tuliptree, *Liriodendron tulipifera*, heavily infested with *T. liriodendri*.

**Figure 2.**
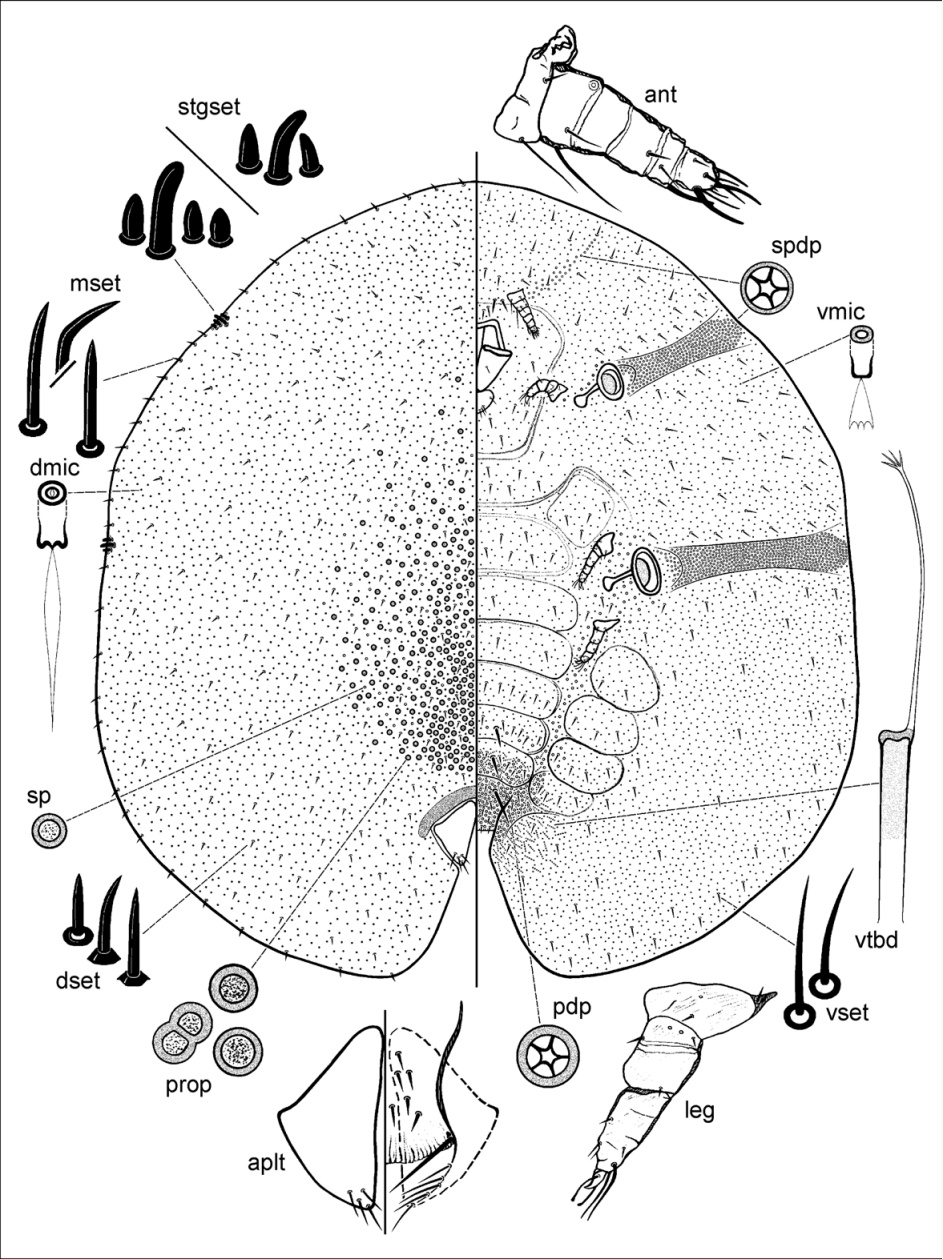
*Toumeyella liriodendri* (Gmelin), adult female. Showing the dorsum on the left and the venter on the right with enlargements of important features around the margin, aplt = anal plate; ant = antenna; dmic = dorsal microduct; dset = dorsal setae; mset = marginal setae; sp = simple pore; pdp = pregenital disc-pore; prop = preopercular pores; spdp = spiracular discpore; stgset = stigmatic setae; vmic = ventral microduct; vset = ventral setae; vtbd = ventral tubular duct.

*Toumeyella liriodendri* (Gmelin) Figures 1, 2
*Coccus liriodendri*; Gmelin 1790: 2220.
*Lecanium tulipifera*; Cook 1878: 192.
*Lecanium liriodendri*; Cockerell 1899: 271.
*Eulecanium liriodendri*; Fernald 1903: 190.
*Lecanium (Toumeyella) liriodendri*; Pettit and McDaniel 1920: 10.
*Toumeyella liriodendri*; Sanders 1909: 447; Burns & Donley 1970: 228; Williams and Kosztarab 1972: 164; Hamon and Williams 1984: 119; Gill 1988: 111; Miller and Williams 1990: 354; Miller and Williams 1995: 16; Ben-Dov 1993: 329; Kosztarab 1996: 391; Sheffer and Williams 1990: 48.
*Common name*: Tuliptree scale, approved by The Entomological Society of America (Stoetzel, 1989).

### Material studied

Neotype, adult female, here designated, 1(1) (USNM). U.S.A., Alabama, Auburn, 32°36′50″N, 85°28′50″W, 2.v.2006, coll. T. Kondo, ex *Liriodendron tulipifera. Other material studied.* Same data as neotype: 4(4 adult females) (BME), 10(10 adult females) (BMNH), 3(3 adult females) (MNNH), 16(15 adult females + 1 3rd instar nymph) (USNM).

## Description. Adult female

### Unmounted material

(Figure 1A & B) Body convex, mid-dorsum elevated, highly convex or flattening towards margin. Derm orange in color, heavily mottled in grayish-blue or grayish green, to dark tessellations, but usually with mid-dorsum very lightly or not mottled. Mature insects 2.6–4.6 (3.0) mm in diameter, and 2.5–4.5 (2.5) mm high.

### Mounted material

(Figure 2) Body outline oval, narrowing anteriorly, often asymmetrical in crowded specimens; body 2.5–4.5 (2.8) mm long, 1.8–4.2 (2.8) mm wide (n=34).

### Dorsum

Derm membranous on both young and older adult females. Dorsal setae (dset) sharply spinose, straight or slightly curved, each 12–18 ***µ*** m long, more or less scattered evenly. Dorsal microducts (dmic) each about 2.5 ***µ*** m wide, with a long terminal filament, evenly scattered. Simple pores (sp) each 3–4 ***µ*** m wide, evenly scattered. Dorsal tubular ducts absent. Preopercular pores (prop) numerous, present on mid-dorsum anterior to anal plates, each pore 6–15 [mostly 10–13] ***µ*** m wide. Dorsal tubercles and pocket-like sclerotizations absent. Anal plates (aplt) together quadrate, with notched round outer angles, plates located at about ⅕ of body length from posterior margin, each plate 235–255 (245) ***µ*** m long, 125–150 (125) ***µ*** m wide, anterolateral margin 155–190 (175) ***µ*** m long, posterolateral margin 170–185 (150) ***µ*** m long, with 4 setae on dorsal surface, plus 1 pair of long fringe setae, about 5 ventral subapical setae and 6 pairs of hypopygial setae. Anal ring with 10 setae (not illustrated). A sclerotic area often present around anal plates on area anterior to anal plates.

### Margin

Marginal setae (mset) sharply spinose, more robust than dorsal setae, straight to strongly bent, each 16–36 ***µ*** m long, arranged in a single, often irregular row, with 7–21 (10 or 11 on neotype) on each side between anterior and posterior stigmatic areas. Stigmatic clefts very shallow or absent, usually with 3 setae per stigmatic area, but often with 4 or rarely 2 setae (4 on each anterior stigmatic cleft and 3 on each posterior stigmatic cleft on neotype); stigmatic setae (stgset) bluntly spinose to conical, all setae subequal in length or with a longer seta, longest seta on each stigmatic area 32–43 ***µ*** m long, other setae 17–30 ***µ*** m long. Eyes not detected.

### Venter

Derm entirely membranous. Pregenital disc-pores (pdp) each 6.0–7.5 ***µ*** m wide, mostly with 5 loculi, rarely 3 or 4, or 6–8 loculi, present around vulvar area and across each posterior abdominal segments (segments IV–VI), with a linear group of pores extending from area around posterior legs to posterior spiracles on each side. Spiracular disc-pores (spdp) each 6–8 (mostly 6–7) ***µ*** m wide, with 5 loculi, rarely a few pores with fewer or more loculi, present in a broad band as wide as peritreme extending laterally from each spiracle to body margin, pore band often narrowing just before reaching margins. Multilocular disc-pores similar to spiracular disc-pores present in a linear group of up to 40 pores extending from each antenna towards body margin, but often absent or very few and restricted to area around antennal scape. Ventral microducts (vmic) scattered evenly throughout, each about 4 × 3 ***µ*** m wide. Ventral tubular ducts present around vulvar region, and anteriorly as far as abdominal segment V, each tubular duct with a terminal filament ending in a small, branched gland. Ventral setae slender, straight or slightly bent, each 12–25 ***µ*** m long; also 3 pairs of long median setae, each 50–115 ***µ*** m long, a pair on segment VI longest. Spiracles well developed, large, anterior spiracular peritremes each 90–175 (140) ***µ*** m wide, posterior peritremes each 110–225 (165) ***µ*** m wide. Legs greatly reduced, but most segments usually discernible, with trochanter and femur, and tibia and tarsus fused, all segments with few setae, total length of all legs: each 125–265 (125–250) ***µ*** m long, metathoracic legs usually largest; claws without a denticle, claw digitules, slender, knobbed; tarsal digitules knobbed or spiniform, as long as or slightly longer than claw digitules. Antennae (ant) short, each 115–190 (153–155) ***µ*** m long, 4–6 (6) segmented, but some specimens with antennae heavily atrophied with segmentation not discernible; often with a very long seta on pedicel, with fleshy setae present on last segment only. With 2 pairs of thick interantennal setae, each 18–50 (20–45) ***µ*** m long, and with 2 pairs of smaller setae just above mouthparts. Mouthparts well developed, clypeolabral shield 235–300 (270) ***µ*** m wide; labium 1 segmented, with 4 pairs of labial setae.

### Morphological variation

The stigmatic setae appear to be a plastic feature. There are usually 3 setae in each stigmatic area, but some specimens often have 4 or fewer setae. The number of multilocular pores near the antennae is also variable, ranging from none or just a few near each antennal scape to about 40 pores in a linear group extending from around antennal scape antero-laterally towards body margin.

### Notes

Measurements of the neotype are given in parentheses. The first-instar nymph of *T. liriodendri* has been described by Sheffer and Williams (1990), the test of the adult male by Miller and Williams (1990), and the adult male by Miller and Williams (1995).

### Editor's Note

Paper copies of this article will be deposited in the following libraries. Senckenberg Library, Frankfurt Germany; National Museum of Natural History, Paris, France; Field Museum of Natural History, Chicago, Illinois USA; the University of Wisconsin, Madison, USA; the University of Arizona, Tucson, Arizona USA; Smithsonian Institution Libraries, Washington D.C. U.S.A.; The Linnean Society, London, England.
